# FEAR index in predicting treatment among patients with femoroacetabular impingement and hip dysplasia and the relationship of femoral version

**DOI:** 10.1093/jhps/hnac023

**Published:** 2022-04-27

**Authors:** Alex M Meyer, Andrew L Schaver, Brian H Cohen, Natalie A Glass, Michael C Willey, Robert W Westermann

**Affiliations:** Carver College of Medicine, University of Iowa, Iowa City, IA 52242, USA; Department of Orthopedics & Rehabilitation, University of Iowa, Iowa City, IA 52242, USA; Orthopedic Associates, Cranston, RI 02910, USA; Department of Orthopedics & Rehabilitation, University of Iowa, Iowa City, IA 52242, USA; Department of Orthopedics & Rehabilitation, University of Iowa, Iowa City, IA 52242, USA; Department of Orthopedics & Rehabilitation, University of Iowa, Iowa City, IA 52242, USA

## Abstract

The Femoro-Epiphyseal Acetabular Roof (FEAR) index is a newer measurement to identify the hip instability with borderline acetabular dysplasia. The purpose of this study is to (i) validate the FEAR index in determining the stability of the hip in patients who have previously been treated surgically for femoroacetabular impingement (FAI) and/or developmental dysplasia of the hip (DDH) and (ii) to examine the relationship between the FEAR index and femoral version, lateral center edge angle, Tönnis angle and alpha angle (AA). Patient demographics and radiographic measurements of 215 hips (178 patients), 116 hips treated with hip arthroscopy for FAI and 99 hips treated with periacetabular osteotomy (PAO) for DDH were compared between groups. The sensitivity and specificity of the FEAR index to detect the surgical procedure performed (PAO or hip arthroscopy) was calculated, and a threshold value was proposed. Pearson’s correlation coefficients were used to describe the relationships between the FEAR index, femoral version and other radiographic measurements. The FEAR index was higher in patients with DDH versus FAI (DDH: 2.81 ± 0.50° versus FAI: −1.00 ± 0.21°, *P* < 0.001). A FEAR index threshold value of 3° had a sensitivity and specificity of 80% and 81%, respectively, for correctly predicting the surgical procedure performed. Femoral version was positively associated with the FEAR index in the setting of DDH (*r* = 0.36, *P* = 0.001) but not FAI (*r* = 0.02, *P* = 0.807). A FEAR index of 3° predicted treatment with 80% sensitivity and 81% specificity. In addition, femoral version significantly correlates with the FEAR index in the setting of DDH but not FAI.

## INTRODUCTION

**Fig 1.**
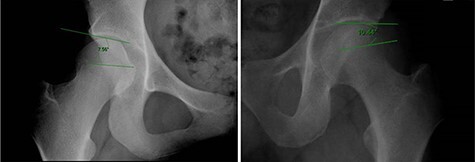
The FEAR index is measured as the angle between lines connecting the medial and lateral edges of the acetabular sourcil and the physeal scar of the femoral head. A laterally diverging angle is considered a positive FEAR index, while a laterally converging angle is considered a negative FEAR index.

Developmental dysplasia of the hip (DDH) is one of the most common causes of hip osteoarthritis [1–3]. The severity of DDH has traditionally been classified according to the lateral center edge angle (LCEA) of Wiberg, which assesses the superolateral coverage of the femoral head by the acetabulum on an anteroposterior (AP) pelvis radiograph [4]. An LCEA of 25–35° is considered normal, 20–25° is borderline dysplasia, 15–20° is mild dysplasia and <15° is moderate-to-severe dysplasia [4, 5]. Treating patients with borderline acetabular dysplasia is particularly challenging, specifically determining whether the patient’s symptoms are due to DDH or femoroacetabular impingement (FAI) [6, 7]. The Femoro-Epiphyseal Acetabular Roof (FEAR) index was recently introduced to help predict the presence of instability in patients with borderline acetabular dysplasia [7–10]. The FEAR index is measured as the angle between lines connecting the medial and lateral edges of the acetabular sourcil and the physeal scar of the femoral head [7]. A laterally diverging angle is considered a positive FEAR index, while a laterally converging angle is considered a negative FEAR index (Fig. 1). Wyatt *et al.* [7] determined that a positive FEAR index cutoff of 5° was 79% accurate in identifying whether a stable or unstable hip was present in a group of patients with borderline hip dysplasia.

Few studies have evaluated the utility of the FEAR index in classifying stability in non-dysplastic hips as well as to evaluate the relationship between the FEAR index and other radiographic measurements of the hip [8, 11]. In particular, femoral version—the amount of rotational deformity of the femur—is often variable in both dysplastic and non-dysplastic hips, but in general, higher than average femoral anteversion is expected in the setting of hip dysplasia [12]. The degree of femoral version can impact the decision to treat borderline dysplastic hips with periacetabular osteotomy (PAO) and/or hip arthroscopy (HA). For example, as described by Chaharbakhshi *et al.*, patients with excessive femoral anteversion and borderline hip dysplasia have poorer post-operative outcomes following HA than patients with normal coverage and femoral version. Therefore, according to their findings, patients with borderline dysplasia and excessive femoral anteversion may better be treated with PAO instead of HA [13]. Measuring the degree of femoral version as well as the FEAR index could potentially help hip surgeons better determine the most appropriate treatment for patients with DDH and/or FAI.

The FEAR index has been introduced to help identify the presence of hip instability in the setting of borderline acetabular dysplasia [10]. It has been shown that femoral version also plays a role in affecting the stability in a non-dysplastic hip [14]. Both the FEAR index and abnormal version have been reported to be associated with hip instability, but the FEAR index’s association with femoral version has not been studied. Therefore, the first purpose of the current study was to validate the FEAR index in determining the stability of the hip in a group of patients who have previously been treated surgically for FAI and/or DDH. The second purpose of this study is to determine a cutoff value for the FEAR index to predict the stability of the hip and to evaluate the relationships between the FEAR index and primarily femoral version, as well as LCEA, Tönnis angle (TA) and alpha angle (AA) in FAI and DDH. Our hypothesis was that the FEAR index would be increased in the setting of DDH versus FAI, and the FEAR index would be positively associated with femoral version in the setting of DDH versus FAI.

## PATIENTS AND METHODS

Approval for this study was granted by the Institutional Review Board. A retrospective review of our institution’s prospectively collected hip preservation registry was performed. Pre-operative pelvic radiographs from patients aged ≤40 years who underwent peri-acetabular osteotomy (PAO) for DDH or HA for FAI between 1 January 2015 and 1 April 2020 were included. Since only pre-operative radiographs were used for data collection, a minimum follow-up requirement was not defined.

**Fig. 2.**
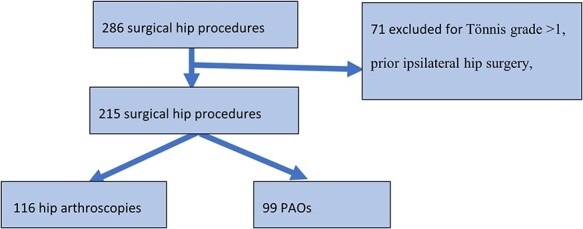
Inclusion and exclusion criteria of the study patients.

Within the study period, all patients who presented to our institution’s young adult hip preservation clinic were evaluated for both FAI and DDH. Patients diagnosed with hip pain and treated with hip preservation surgery were enrolled in the hip preservation registry. A standardized evaluation of patient history, physical exam and radiographic findings on AP pelvis and pelvic computed tomography (CT) scans were used to diagnose both conditions. All AP pelvic radiographs were obtained in the standing position using a standard protocol using the same X-ray machine and X-ray technicians. Patients with FAI syndrome were diagnosed in accordance with the Warwick Agreement [15], and all patients received arthroscopic surgery to address associated cam or pincer morphology. AP and false-profile views of the pelvis and hip were used to evaluate for signs of instability, including a decreased LCEA of Wiberg and an elevated TA [16, 17]. Patients diagnosed with DDH were surgically treated with PAO. Those with elevated or decreased femoral anteversion treated with femoral de-rotational osteotomy were not included as the measured rotation in our study would not be their native rotation. Exclusion criteria for the study consisted of a Tönnis grade of >1, prior ipsilateral hip surgery, revision surgeries, or residual deformities (post-slipped capital femoral epiphysis or Perthes). In the selected time frame, there were 286 surgeries performed by 2 hip surgeons, 146 underwent HA for FAI, 140 underwent PAO, 30 were excluded in the FAI group and 41 were excluded in the DDH group as per the pre-determined exclusion criteria outlined above. Overall, this study includes 215 operative hips (178 patients), including 116 hips treated with HA for FAI (99 patients) and 99 hips treated with PAO for DDH (79 patients) (Fig. 2).

Values for the FEAR index, femoral version, LCEA, TA and AA on the AP pelvis, Dunn lateral and frog-leg lateral orientations were recorded. The FEAR index was measured as the angle between two lines on the AP pelvis radiograph: one connecting the medial and lateral portions of the femoral physeal scar and one connecting the medial and lateral points of the sclerosis of the sourcil. A laterally diverging angle was considered a positive FEAR index as previously described [7], and femoral version is defined as the angle between the femoral head/neck and the distal femoral condyles [18]. We measured the femoral version using the angle of the femoral head/neck by utilizing a reformatted axial oblique full-limb CT scan, which has been demonstrated to be more accurate in determining the amount of femoral anteversion [19] and the distal femoral intercondylar axis. The LCEA was measured as the angle between the vertical axis of the pelvis and a line connecting the center of the femoral head and the most lateral point of the acetabular sourcil [4]. TA and AA were measured as previously described [4]. TA was measured on the AP view, while the AA was measured on AP, Dunn and frog-leg lateral views. Pre-operative pelvic radiographs and CT scans were analyzed by two independent reviewers, an orthopedic sports medicine fellow and medical student (B.H.C. and A.M.M.), and intra-class correlation coefficients (ICCs) were determined to evaluate inter-rater reliability (Table I).

**Table I.** Intra-class correlation coefficients

*Measure*	*Intra-class correlation coefficients*
Femoral version	0.95 (0.94–0.96)
FEAR index	0.88 (0.85–0.90)
LCEA	0.94 (0.93–0.95)
TA	0.95 (0.94–0.96)
AP AA	0.92 (0.90–0.93)
Dunn AA	0.95 (0.93–0.95)
Frog-leg AA	0.95 (0.94–0.96)

Participants were defined as having unstable (DDH group) or stable (FAI group) hips and grouped accordingly. These patients were classified by the surgical procedure that they had performed, and it is assumed that the correct surgical procedure was performed. The indications for PAO were an LCEA of <20° or an LCEA of 20–25° with symptoms of instability. The proportion (percentage) of female participants and mean ± SD of age and radiographic measures were determined and compared between groups using the chi-squared test or *t*-tests, respectively. Potential between-group differences in radiographic measures were also assessed using generalized linear models that accounted for correlation between hips within subjects.

Pearson’s correlation coefficients were used to describe the relationships between the femoral version, TA, LCEA and FEAR index for participant-level analyses. The correlation coefficient for repeated measures was also calculated for hip level analyses [20]. The inter-observer reliability in the FEAR index, LCEA, TA, AP AA, Dunn AA and frog-leg AA measurements were described using ICCs. Receiver operating characteristic (ROC) curves analyses were used to determine the area under the curve (AUC) for the FEAR index to detect treatment with PAO. The threshold FEAR index value for detecting PAO was determined using the Youden index, a method that determines the point on the ROC curve that maximizes sensitivity and specificity. A *P*-value of <0.05 was considered statistically significant.

## RESULTS

### Validation of the FEAR index, femoral version in DDH and FAI

**Fig. 3.**
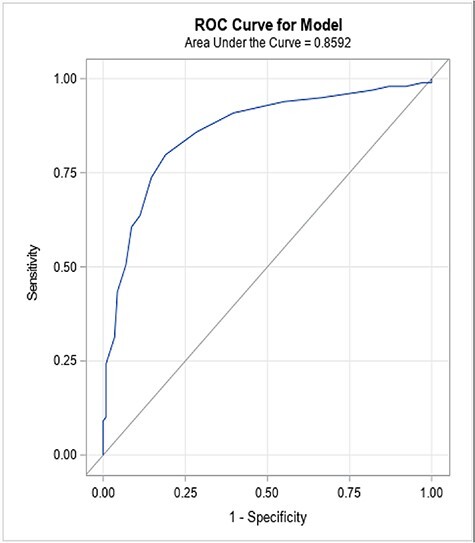
Sensitivity and specificity of using a FEAR index of 3° as a predictor of instability. AUC = 0.86.

Of the 215 operative hips, consisting of 178 patients, including 116 hips treated with HA for FAI (99 patients) and 99 hips treated with PAO for DDH (79 patients), the mean age did not significantly differ between groups (FAI 24.5 ± 7.1 versus DDH 23.1 ± 7.8 years, *P* = 0.207). However, the percentage of males treated was significantly higher in the FAI group [FAI 39 (39.4%) versus DDH 8 (10.1%) patients, *P* < 0.001) (Table II). The mean FEAR index was significantly more positive in the DDH group (DDH 3.01° ± 0.50° versus FAI −0.77° ± 0.21°, *P* < 0.0001), while femoral version was not significantly different between groups (DDH 15.81° ± 1.42° versus FAI 13.92° ± 1.09°, *P* = 0.268). The FEAR index was found to be reproducible among researchers with an intra-class correlation coefficient of 0.88 as was femoral version (0.95), LCEA (0.94), TA (0.95), AP AA (0.92), Dunn AA (0.95) and frog-leg AA (0.95) (Table I). The remaining comparisons are presented in Table II. Using the Youden index to select a threshold value of the FEAR index to predict treatment, we found that a FEAR index of >3° had a sensitivity and specificity of 80% and 81%, respectively, for predicting treatment with PAO. A FEAR index of ≥3° was associated with significantly increased odds of receiving PAO for DDH (odds ratio (OR) = 17.05, 95% confidence interval (CI) = 6.26–46.90, *P* < 0.0001) (Fig. 3).

**Table II.** Differences in age, gender distribution and radiographic measures in FAI compared with DDH

	*FAI* *N = 99 participants* *(n = 116 hips)*	*DDH* *N = 79 participants* *(n = 99 hips)*	*P-value*
Mean age(years, mean ± SD)	24.5 ± 7.1 (24.3 ± 7.0)	23.1 ± 7.8 (22.9 ± 7.8)	0.2070 (0.2117)
Males (%)	39 (39.4%)	8 (10.1%)	<0.0001 (<0.0001)
FEAR index(units, mean ± SD)	−0.77 ± 0.21 (−1.00 ± 0.21)	3.01 ± 0.50 (2.81 ± 0.60)	<0.0001 (<0.0001)
Femoral version(units, mean ± SD)	13.92 ± 1.09 (13.92 ± 0.89)	15.81 ± 1.42 (16.22 ± 1.57)	0.2677 (0.1945)
LCEA(degrees, mean ± SD)	31.49 ± 0.65 (31.61 ± 0.49)	21.24 ± 0.85 (21.43 ± 0.88)	<0.0001 (<0.0001)
TA(degrees, mean ± SD)	5.81 ± 0.47 (5.60 ± 0.28)	14.59 ± 0.61 (13.93 ± 0.71)	<0.0001 (<0.0001)
AA—AP(degrees, mean ± SD)	52.15 ± 0.44 (52.05 ± 0.53)	51.56 ± 057 (51.60 ± 0.95)	0.3881 (0.6153)
AA—Dunn	60.93 ± 0.76 (60.67 ± 0.70)	62.89 ± 0.99 (63.22 ± 1.00)	0.0972 (0.0364)
AA—Frog	54.24 ± 0.62 53.87 ± 0.64	55.17 ± 0.82 (55.17 ± 1.02)	0.3426 (0.2083)

Differences in age, gender distribution and radiographic measures in FAI compared with DDH with statistically significant results are in **bold**.

### Correlation of the FEAR index with femoral version

**Fig. 4.**
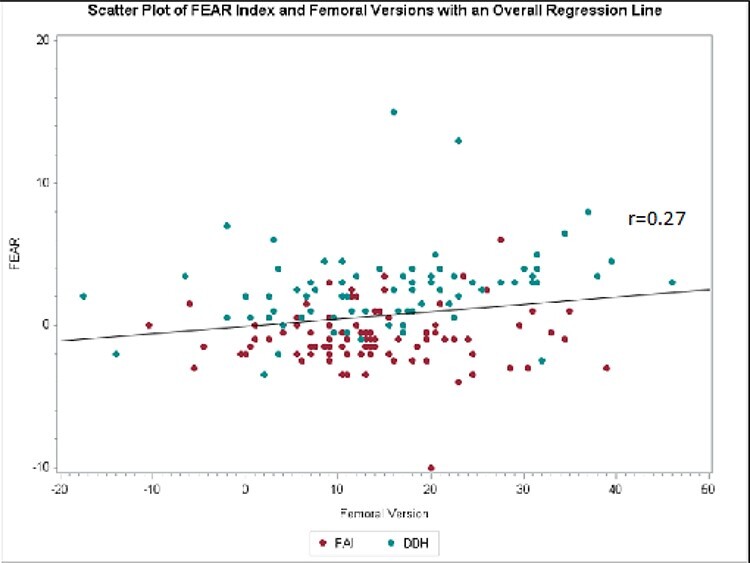
Correlation between femoral version and the FEAR index.

The FEAR index significantly correlated with femoral version in patients with DDH (*r* = 0.36, *P* = 0.001) but not in patients with FAI (*r* = 0.02, *P* = 0.807) (Table III). That is, although patients with DDH did not have a greater average femoral version than patients with FAI, those in the DDH group with a higher FEAR index were also found to have higher degrees of femoral anteversion (Fig. 4). The remaining correlations for LCEA and TA are listed in Table II. All caluclations were adjusted for gender as there were different proportions of males and females in each study group.

**Table III.** Pearson’s correlation coefficients for the relationships between the FEAR index and femoral version, LCEA and TA, and FAI and DDH

	*All N = 178* *participants* *(n = 215 hips)*	*FAI N = 99* *participants* *(n = 116 hips)*	*DDH N = 79* *participants* *(n = 99)*
Femoral version	0.25, *P* = 0.0007 (0.27)	0.02, *P* = 0.8074 (0.07)	0.36, *P* = 0.0011 (0.34)
LCEA	−0.80, *P* < 0.0001 (−0.66)	−0.34, *P* = 0.0006 (−0.26)	−0.83, *P* < 0.0001 (−0.62)
TA	0.76, *P* < 0.0001 (0.65)	0.43, *P* < 0.0001 (0.34)	0.72, *P* < 0.0001 (0.55)

## DISCUSSION

The validity of the FEAR index has been evaluated in the setting of borderline acetabular dysplasia, but the use of the FEAR index in classifying stability of dysplastic and non-dysplastic hips by predicting the surgical procedure performed has not been previously studied. In addition, the correlation between the FEAR index and femoral version has not been well established. The results of this study demonstrate that using a FEAR index of 3° has 80% sensitivity and 81% specificity for predicting the treatment of PAO for DDH. The results also demonstrated femoral version is significantly associated with the FEAR index in DDH but not FAI, illustrating the role femoral version plays in the stability of an FAI hip. Our data suggest that abnormal femoral version alone does not predict instability, but abnormal version in combination with a higher FEAR index likely are both contributing factors to hip instability. This previously was demonstrated by Chaharbakhshi *et al.* who found that patients with excessive femoral anteversion and borderline dysplasia had worse post-operative outcomes following HA than patients with normal version and without borderline dysplasia [13] in the setting of FAI. Adding to the literature, our results suggest that it may play a larger role in DDH, which has not been demonstrated in any previous studies. One potential explanation of these differences is that cam morphologies could potentially be protective by preventing instability of the hip due to decreasing range of motion in hip flexion and internal rotation [14].

The results of our study demonstrate that the FEAR index is significantly higher in patients with hip dysplasia versus FAI syndrome. In addition to LCEA and TA, our data suggest that a larger FEAR index is positively associated with the treatment of hip instability with PAO. Previously, LCEA and TA were the main measurements for evaluating the stability of the hip, but recently, Wyatt *et al.* introduced the FEAR index as a novel measurement for predicting instability in a symptomatic borderline dysplastic hip [7]. Our results had a sensitivity of 80% and specificity of 81% for predicting surgical treatment when using a FEAR index cutoff of 3°. This is compared with Wyatt *et al.* reporting a sensitivity of 78% and specificity of 80% for predicting instability when using a FEAR index threshold of 5° [7], Batailler *et al.* who reported 90% accuracy when using a FEAR index of <2° [10] and Truntzer *et al.* who reported 92.4% specificity of predicting an unstable hip when the FEAR index was >5° [8]. The primary outcome of this study was the surgery performed. As we demonstrated with our results, the FEAR index was able to accurately predict this outcome. Therefore, our results have further demonstrated that the FEAR index is a reliable tool for predicting treatment rendered.

The findings of this study also demonstrate that femoral version is significantly correlated with the FEAR index in the setting of DDH. This is the first study to evaluate the relationship between femoral version and the FEAR index. The degree of femoral version impacts the decision to treat a borderline dysplastic hip with either HA or PAO. One study [13] found that patients with borderline dysplasia and high degrees of femoral anteversion had significantly lower clinical outcome scores and higher re-operation rates. In a patient with an elevated femoral version, the FEAR index should be measured to evaluate for instability, as it has now been shown to correlate with an elevated FEAR index. This furthers the utility of this new measurement and may offer insight into the relationship between femoral version, hip instability and DDH. For example, in regard to a borderline dysplastic hip with high femoral anteversion, a surgeon may consider de-rotational osteotomy as opposed to HA in order to improve post-operative outcomes.

This study was limited by being a single-institution study using cases from two hip surgeons. Therefore, in order to perform this study, the surgery performed was the primary outcome, and we had to make the assumption that all cases of FAI and/or DDH were treated appropriately with the correct surgical procedure. We used the surgery performed as the primary outcome instead of post-operative outcomes due to the many confounding variables that contribute to post-operative outcomes, including but not limited to the quality of correction performed, amount of correction necessary, prior functional status and age. In an effort to more confidently assume the correct procedure was performed, we included all patients who underwent PAO or HA as opposed to only including borderline dysplastic hips as there is currently debate and no gold standard for the treatment of these hips, which is also a primary reason to perform this study. Additionally, since a hip preservation registry was used, the reviewers were not blinded to diagnosis at the time of data collection; however, potential bias was limited by excellent ICC values between the two primary observers, and having a third reviewer resolves any significant differences (Table I). Our sample size also limited our ability to match the groups more precisely for similar age, sex and body mass index.

In conclusion, this study has demonstrated that high femoral version is positively associated with high FEAR index measurements in the DDH population. We have corroborated previous findings that the FEAR index has good sensitivity and specificity for predicting surgical treatment with PAO. These findings suggest that it is imperative to evaluate femoral version during the workup and operative planning for patients with a symptomatic hip. Clinically, these findings are important when evaluating symptomatic hips for DDH or FAI. Femoral version should also be measured in addition to traditional radiographic measurements. With an improved understanding of the contributing factors for hip instability, we can better address surgically leading to superior outcomes for patients. Future areas of study include cadaveric studies to more precisely evaluate instability of the hip compared with the measured FEAR index, further assessment of the FEAR index in regard to borderline dysplastic hips and also incorporating patient outcomes.

## Data Availability

Data available on request.
